# Current Status on Biochemistry and Molecular Biology of Microbial Degradation of Nicotine

**DOI:** 10.1155/2013/125385

**Published:** 2013-12-29

**Authors:** Raman Gurusamy, Sakthivel Natarajan

**Affiliations:** Department of Biotechnology, School of Life Sciences, Pondicherry University, Puducherry 605014, India

## Abstract

Bioremediation is one of the most promising methods to clean up polluted environments using highly efficient potent microbes. Microbes with specific enzymes and biochemical pathways are capable of degrading the tobacco alkaloids including highly toxic heterocyclic compound, nicotine. After the metabolic conversion, these nicotinophilic microbes use nicotine as the sole carbon, nitrogen, and energy source for their growth. Various nicotine degradation pathways such as demethylation pathway in fungi, pyridine pathway in Gram-positive bacteria, pyrrolidine pathway, and variant of pyridine and pyrrolidine pathways in Gram-negative bacteria have been reported. In this review, we discussed the nicotine-degrading pathways of microbes and their enzymes and biotechnological applications of nicotine intermediate metabolites.

## 1. Introduction 

Tobacco (*Nicotiana*, Solanaceae family) is mainly cultivated in Brazil, China, Cuba, India, and USA. An annual production of 6.7 million tonnes of tobacco has been reported. China is the largest tobacco producer (39.6%) followed by India (8.3%), Brazil (7.0%), and the USA (4.6%) [1]. India is the third largest tobacco consumer (275 million) in the world followed by China and USA [2]. It is anticipated that the tobacco industry would produce 3,00,274 tonnes of nicotine wastes every year [3]. Nicotine usually accounts for more than 90% of the whole plant alkaloid fraction in commercial tobacco, *Nicotiana tabacum* [4]. The entire or part of the tobacco leaf was used as raw material for tobacco products such as cigarette, cigars, chewing tobacco, and snuff.

In the year 2000, it was estimated that 4.9 million deaths occur due to smoking [5]. By the year 2020, it is also expected to exceed 9 million deaths annually [5]. Due to the increased usage of tobacco products, the industry generated solid and liquid tobacco wastes containing high concentrations of nicotine [6]. The tobacco industries produce tobacco waste with an average content of nicotine of 18 g per kg of dry weight [7]. The Environmental Protection Agency (EPA) has classified these nonrecyclable powdery tobacco wastes as Toxic Release Inventory (TRI) chemicals [8]. When the concentration of nicotine content exceeds more than 0.05% (w/w), it is designated as “toxic and hazardous” by the European Union Regulations (EUR) [7]. These tobacco wastes are dumped on the ground without proper storage and processing [7, 8]. Nicotine dissolves easily in water leading to the contamination of the ground water [7, 8]. Hence, the nicotine-contaminated water disturbs the ecological balance of soil [7, 8]. Therefore, it is important to remove nicotine from tobacco polluted soil and water [7, 8].

## 2. Nicotine

Nicotine 3-(1-methyl-2-pyrrolidinyl) pyridine is a heterocyclic compound with a pyridine and a pyrrolidine ring moiety. Nicotine is a pale yellow to dark brown liquid with slight fishy odor when warm. It is water-soluble. The chemical formula of nicotine is C_10_H_14_N_2_. Its molecular weight is 162.234 with melting point –79°C and boiling point 247°C. Nicotine is present up to 2 to 8% of the dry mass of the tobacco leaves [9].

### 2.1. Effect of Nicotine on Human Beings

Nicotine is a hazardous compound that causes tobacco related lung cancer and peripheral arterial disease [10]. Although more than 4000 substances are present in the tobacco cigarette smoke, nicotine is the major substance [11, 12]. Nicotine has a blood half-life period of approximately 2 h and causes severe vascular diseases [11, 12]. Nicotine can cause cancer, gene mutation, and malformation [13]. An array of toxic nicotine intermediate metabolites such as *N*′-nitrosonornicotine, 4-(methylnitrosamino)-1-(3-pyridyl)-1-butanone, cotinine, and *N*-nitrosamine causes tobacco lung cancer [14, 15]. Neurotoxin developmental effects of nicotine can naturally affect a variety of cellular processes such as generation of oxidative radicals, apoptosis, and hyperplasia of cell, enhancing gene expression to secrete hormones and regulation of enzymatic activity [13, 16, 17]. The lungs rapidly absorb 90% of the nicotine that is present in the cigarette smoke inhaled by human beings [18]. Nicotine in the human body can easily pass the biological membranes and blood-brain barrier and increases heart rate, mean arterial blood pressure and mimics the venous endothelial dysfunction [15, 19, 20].

Nicotine is an additive substance that can lead to nicotine dependence and addictive behavior in human populations [43]. Nicotine dependence is complex, multidimensional trait that involves psychological, physiological behavioral, and social factors [44]. Nicotine, the primary psychoactive ingredient of tobacco, contributes to physical dependence, by acting on nicotinic acetylcholine receptors in the central nervous system and leads to the release of neurotransmitters (e.g., dopamine and serotonin), which produce reinforcing effects by activating the mesocorticolimbic dopamine system [45].

## 3. Microbial Degradation of Nicotine

Physical and chemical methods are available to degrade nicotine in the tobacco. These methods are often time-consuming, expensive and involve solvent extraction procedures [46]. Bioremediation is one of the promising methods to clean up polluted environments using microbes [47–49]. Briski et al. [50] reported that aerobic composting is an effective method to reduce 80% of nicotine and 50% of the volume and mass of tobacco solid wastes in 16 d. Meher et al. [51] used a technique called biomethanation that removed 60% of nicotine, 75.6% of chemical oxygen demand, and 80% of biological oxygen demand from the tobacco wastes. Biological method employs a variety of nicotine-degrading bacteria and fungi [41, 46, 51]. These ecofriendly biological methods are extensively used in wastewater treatment due to its high efficiency and low cost. Microbes that degrade nicotine are reported to adapt to polluted environment easily [52, 53]. Native strains of bacteria and fungi that live in the tobacco environment have the ability to degrade nicotine [54]. These microbes utilize nicotine as sole carbon, nitrogen, and energy source for their growth [6]. Batham [55] reported that the nitrate content of the soil increased due to the microbial degradation of nicotine.

### 3.1. Optimal Conditions of Nicotine-Degrading Bacteria

Nicotine degradation experiments are carried out with different culture media such as distilled water, minimal salt medium, inorganic salt medium, and basal salt medium (BSM). The culture media influence the nicotine degradation efficiency. Wang et al. [28] reported that 3 g/L nicotine was fully degraded in 5 h when the degradation experiment was carried out with 0.05 M sodium phosphate buffer (pH 7.0). However, it took more than 8 h to complete degradation when carried out with distilled water. The optimal culture conditions and nicotine degradation efficiency of nicotinophilic bacteria vary from each other. Most of the bacteria grow at 30°C, pH range of 6.4 to 7.5, and degrade maximum concentration of nicotine up to 6 g/L [2, 5]. Huang et al. [56] reported that the rate of nicotine degradation efficiency was high at 37°C when compared to 30°C. The optimal conditions and nicotine degradation efficiency of various nicotine-degrading bacteria are presented in Table 1.

### 3.2. Effect of Trace Elements, Carbon, and Nitrogen Sources on Nicotine Degradation

Trace elements and other carbon and nitrogen sources play a significant role in the biodegradation of nicotine [26]. The presence of (NH_4_)_2_SO_4_ in the nicotine containing medium could decrease the nicotine degradation efficiency [26]. The nicotine degradation rate was increased in the presence of yeast extract, glucose, and Tween 80 in tobacco waste extract (TWE) containing media [57]. However, the nicotine degradation rate was dependent on the concentration of yeast extract and Tween 80 in the TWE medium [27]. In contrast, the medium containing glucose and (NH_4_)_2_SO_4_ reduced the efficiency of nicotine degradation [32]. ZnSO_4_·7H_2_O and NiCl_2_·6H_2_O had no influence on nicotine degradation, whereas Na_2_MoO_4_ and CuCl_2_·4H_2_O inhibited the rate of nicotine degradation [39]. Glucose is an important carbon source that promotes bacterial cell growth and improves the rate of nicotine degradation [24, 34, 39]. The concentration of glucose above 10 g/L inhibited the nicotine degradation by *Pseudomonas *sp. ZUTSKD [34]. Raman et al. [2] reported that 1 g/L dextrose increased nicotine degradation rate. In contrast, glucose is not influenced in the nicotine degradation rate when the experiment was carried out in solid-state fermentation process [39, 56]. Jiang et al. [36] reported that the nicotine degradation rate was slow in the presence of glucose. Conversely, *P. stutzeri *ZCJ could not utilize carbon sources such as sucrose and maltose and nitrogen source, namely, NaNO_2_, for their growth which in turn inhibits nicotine degradation [39].

### 3.3. Effect of Nicotine-Degrading Bacteria on Tobacco Leaves and Tobacco Wastes

Nicotine-degrading bacteria played an important role in the improvement of quality of tobacco leaves during aging (fermentation) process. These bacteria accumulated more in younger tobacco leaves when compared to aged leaves [56]. The desirable flavor, taste, and smoking properties of tobacco remained unaltered when treated with nicotinophilic *Pseudomonas* [6, 29]. Li et al. [42] reported that *Arthrobacter* sp. successfully reduced 28 to 39% of nicotine during flue curing of tobacco leaves and enhanced the quality of tobacco leaf. The nicotine degradation efficiency of different nicotine-degrading bacteria on tobacco leaves and tobacco wastes is listed in Table 2.

The two major bacterial species that degrade nicotine are *Pseudomonas* and *Arthrobacter* [6]. Furthermore, recently, a new nicotine-degrading strain, *Arthrobacter* sp. strain M2012083, was sequenced by Illumina High-Seq 2000, which represented the first sequenced nicotine-degrading *Arthrobacter* strain [58]. Tang et al. [59] recently sequenced a novel bacterial strain *Pseudomonas geniculata* N1 which represents the first sequence of the *Pseudomonas* geniculate group.

### 3.4. Pigment Production during Nicotine Biodegradation

During nicotine degradation different colours were produced. The Gram-positive bacteria *A. nicotinovorans* utilized nicotine and changed to yellow pigments and further turned carmine during nicotine degradation [60–62]. In contrast, Hylin [23] reported that *A. nicotinovorans* did not produce any pigments during nicotine degradation. *Arthrobacter* sp. produced blue violet colour during nicotine degradation due to the transformation of nicotine into 2,3,6-trihydroxypyridine. The colour change was due to the presence of oxygen and the absence of metabolic catalyzing enzymes [63, 64]. The hydroxylated pyridine ring dimerized and formed a blue pigment during nicotine degradation by *Arthrobacter *sp. [62]. Interestingly, during nicotine degradation the Gram-negative bacteria such as *Pseudomonas *sp., *Shinella* sp. HZN1, and *Acinetobacter *sp. ND12 changed the colour of the nicotine medium to green and oxidized to blue gray and finally to brown [2, 26, 31, 32, 36, 42, 57]. The formation of green colour was mainly dependent upon the concentration of nicotine [2, 32, 36]. *P. geniculata* N1 produced golden yellow pigment on the plate during nicotine degradation [59]. However, no pigment was observed during nicotine degradation by *P. putida *and *Ensifer *sp. N7 [30, 33].

### 3.5. Microbial Degradation Procedure

Standard biodegradation methods were employed to treat tobacco wastes [30]. Initially, the tobacco wastes are sterilized under UV light. The resting cells of biodegrading strain are inoculated into tobacco wastes. Then the tobacco wastes are baked at 60°C for 6 days, crushed to a powder, and passed through a sieve with muslin cloth. The tobacco powder is extracted with methanol and the concentration of nicotine is determined as described in [30].

## 4. Biochemical Pathways That Mediate Nicotine Biodegradation

Biodegradation of nicotine by *A. nicotinovorans* and *A. oxidans *has been reported [65, 66]. These Gram-positive bacteria followed pyridine pathway. The nicotine intermediate metabolites of pyridine pathway were identified and characterized [67]. The microbial degradation of nicotine differs among the different strains of species [54]. Previous research suggests that the encoding genes that mediate nicotine degradation are not only located on the bacterial chromosomes but also present in the plasmids [65, 66, 68]. Plasmid-borne genes (160 Kb) in *A. nicotinovorans* are responsible for nicotine degradation [65, 66]. Similarly, nicotine-degrading genes are located on the outside of the chromosomes of *P. convexa* [68]. The bacteria such as *Arthrobacter* sp. (Gram-positive) followed pyridine pathway, which attacked the pyridine ring of the nicotine during degradation. The Gram-negative bacteria *Pseudomonas* sp. initially attacked pyrrolidine ring and followed pyrrolidine pathway. Fungi employed the demethylation pathway that demethylates methyl group in the pyrrolidine ring of the nicotine. Surprisingly, *Agrobacterium* sp. followed a new variant pathway of pyridine and pyrrolidine for nicotine degradation. Several nicotine-degrading genes have been reported [6, 45, 69].

### 4.1. Pyridine Pathways of Nicotine Biodegradation

**Figure 1:**
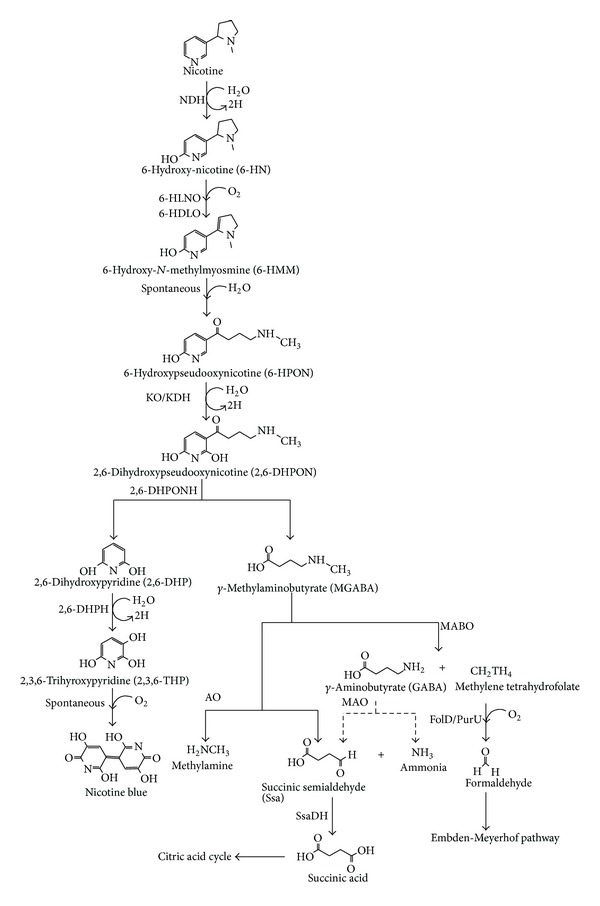
The pyridine pathway of nicotine degradation by *A. nicotinovorans* and *Nocardioides *sp. NDH, nicotine dehydrogenase; 6-HLNO, 6-hydroxy-L-nicotine oxidase; 6HDNO, 6-hydroxy-D-nicotine oxidase; KO, ketone oxidase; KDH, ketone dehydrogenase; 2,6-DHPONH, 2-6-dihyroxypseudooxynicotine hydrolase; 2,6-DHPH, 2,6-dihydroxypyridine-3-hydroxylase; MABO, *γ*-*N*-methylaminobutyrate oxidase; MAO, monoamine oxidase; AO, amine oxidase; FolD, methylenetetrahydrofolate dehydrogenase/cyclohydrolase; PurU, formyltetrahydrofolate deformylase; SsaDH, succinic semialdehyde dehydrogenase.

Pyridine pathways of nicotine degradation by *A. nicotinovorans*, *Nocardioides* sp. JS614 are presented in Figure 1. *A. nicotinovorans* harbors a 160 Kb plasmid which is responsible for nicotine degradation [65, 66]. These bacteria initially attacked 6th position of carbon in the pyridine ring of nicotine by hydroxylation to introduce hydroxyl group and formed 6-hydroxynicotine (6-HN). This hydroxylation step was catalyzed out by nicotine dehydrogenase (NDH). It is a heterotrimeric molybdenum enzyme called nicotine : accepter oxidoreductase [70–73]. This NDH is a heterotrimeric enzyme of xanthine dehydrogenase family containing dinucleotide form of molybdopterin cofactor, a flavin adenine dinucleotide (FAD), and two iron-sulphur clusters [74]. This 6-HN was oxidized to form an optically inactive 6-hydroxy-*N*-methlymyosmine (6-HMM) by oxidation of pyrrolidine ring at the 2nd position of carbon. Interestingly, this step was catalyzed by two enzymes, namely, 6-hydroxy-L-nicotine oxidase (6-HLNO) and 6-hydroxy-D-nicotine oxidase (6HDNO). The dimeric form of 6-HLNO and monomeric form of 6HDNO contained FAD noncovalently bound to 47 kDa and covalently to 49 kDa polypeptide, respectively [75]. The third step was followed by hydration of 6-HMM that spontaneously leads to the opening of the pyrrolidine ring and tautamerization of ketone moiety to form 6-hydroxypseudooxynicotine (6-HPON) [76]. The 2nd position of the carbon of pyridine ring of this metabolite was further hydroxylated by ketone oxidase or ketone dehydrogenase enzyme similar to nicotine dehydrogenase to form 2,6-dihydroxypseudooxynicotine (2,6-DHPON) [77]. The subsequent cleavage of side chain of 2,6-DHPON to form *γ*-*N*-methylaminobutyrate (MGABA) and 2,6-dihydroxypyridine (2,6-DHP) by the action of 2-6-dihyroxypseudooxynicotine hydrolase (2,6-DHPONH) [61, 78–80]. 2,6-DHP was further hydroxylated to form 2,3,6-trihydroxypyridine (2,3-6-THP) by the addition of hydroxyl group to the 3rd position of carbon in the pyridine ring. This step was catalyzed by an enzyme 2,6-dihydroxypyridine-3-hydroxylase (2,6-DHPH) in a strictly nicotinamide adenine dinucleotide hydrogen (NADH)-dependent manner. Dimeric flavoprotein of this enzyme is tightly bound noncovalently to a FAD subunit and inhibited by 2,3-dihydroxypyridine and 2,6-dimethoxypyridine. Each subunit consists of 397 amino acids and mass of 43.4 kDa with addition of one FAD molecule [81]. In the presence of oxygen, spontaneous oxidation of 2,3,6-THP and dimerization of this hydroxylated pyridine ring moiety formed nicotine blue colour [63]. However, Sachelaru et al. [82] reported that *mobA* gene that encodes MobA protein with molybdenum cofactor cytidylate transferase was responsible for formation of nicotine blue colour in *A. nicotinovorans*.

The degradation of MGABA was regulated by cluster of genes encoded by plasmid pAO1. This cluster of genes contained *purU*-*mabO*-*folD* operon that transcribed only in the presence of nicotine and was regulated by transcriptional activator pmfR [83]. MGABA was further degraded into two pathways. The first pathway started with an enzyme *γ*-*N*-methylaminobutyrate oxidase (MABO), which catalyzed MGABA to form *γ*-aminobutyrate (GABA) and methylenetetrahydrofolate. The methylene group of methylenetetrahydrofolate was further oxidized by two enzymes, namely, methylene-tetrahydrofolate dehydrogenase/cyclohydrolase (FolD) and formyltetrahydrofolate deformylase (PurU) to formaldehyde [77]. These two enzymes are nicotinamide adenine dinucleotide hydrogen phosphate (NADPH) dependent and formaldehyde may be assimilated in the Embden-Meyerhof pathway [84, 85]. Another metabolite, GABA metabolized into an ammonia and succinic semialdehyde (Ssa) by monoamine oxidase (MAO), is an amine oxidase (AO) enzyme family. Ssa is further oxidized to succinic acid by an enzyme called nicotinamide adenine dinucleotide phosphate (NADP^+^) dependent succinic semialdehyde dehydrogenase (SsaDH) [86, 87]. This succinic acid entered into citric acid cycle and this catabolic pathway of *γ*-aminobutyrate was normally found in bacteria [88–90]. A newly discovered second degradation pathway of MGABA is deaminated into Ssa and methylamine by amine oxidase (AO) with reduction of FAD to FADH_2_. It produced succinic acid, which entered citric acid cycle [83, 91].

### 4.2. Pyrrolidine Pathways of Nicotine Biodegradation

The extrachromosomal genes of* Pseudomonas* sp. that degrades organic pollutants such as octane, camphor, toluene, methyl benzoate, salicylate, naphthalene, and xylene are responsible for nicotine degradation [68]. A purple crystalline substance, *N*-methylmyosmine, was isolated from nicotine containing medium during the degradation of nicotine by *A. oxydans* [92, 93]. The transformation of intermediate metabolites of nicotine such as 3-nicotinoylpropionic acid, pseudooxynicotine, and *N*-methylmyosmine produced by *Pseudomonas *sp. has been reported [25, 94–96]. Thacker et al. [68] reported that *P. convexa *Pc1 degraded nicotine to 2,5-dihydroxypyridine (2,5-DHP) via pseudooxynicotine, 3-succinoyl pyridine (SP), and 6-hydroxy-3-succinoyl pyridine (HSP) by nicotine-degrading enzyme 2,5-dihydroxypyridine oxygenase. *P. plecoglossicida *TND35 degraded nicotine to 4-hydroxy-1-(3-pyridyl)-1-butanone (HPB) via *N*-methylmyosmine [2].

**Figure 2:**
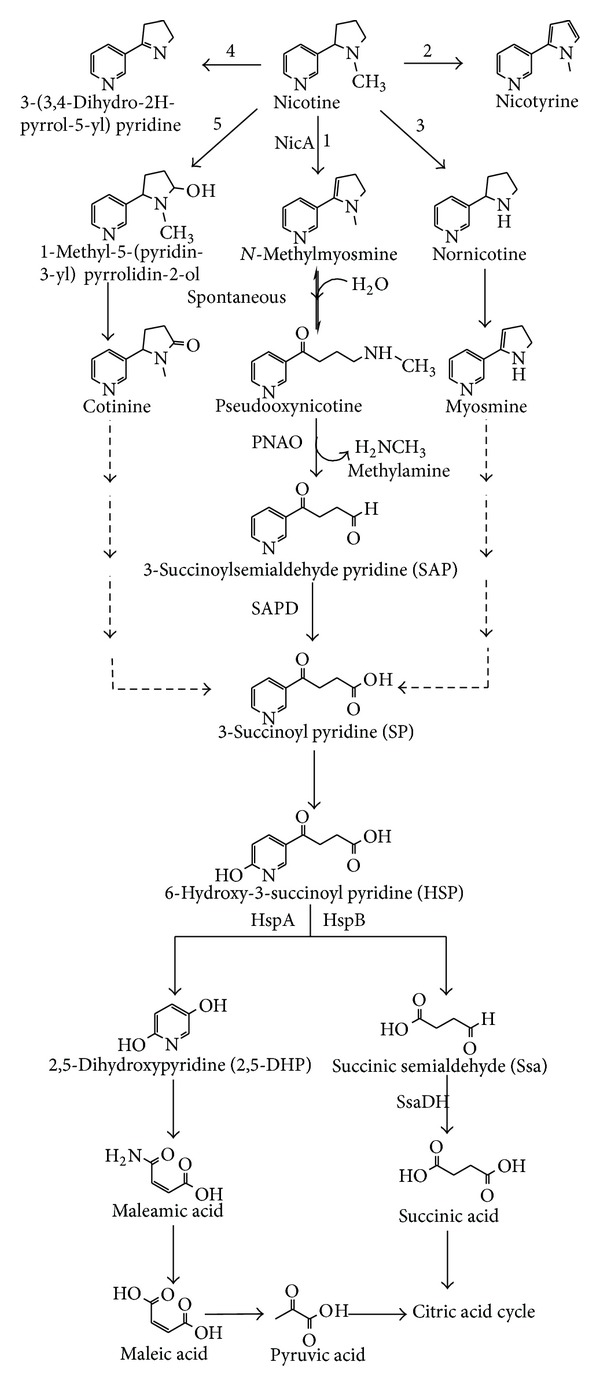
Various pyrrolidine pathways of nicotine degradation followed by 1. *Pseudomonas *sp. HZN6 and *P. putida *S16; 2. *Pseudomonas* sp. HF-1; 3. *Pseudomonas* sp. HF-1 and *Pseudomonas *sp. Nic22; 4. *Pseudomonas* sp. HF-1, *Pseudomonas *sp. Nic22, and *Pseudomonas *sp. CS3; 5. *Pseudomonas *sp. CS3. NicA, nicotine oxidoreductase; PNAO, pseudooxynicotine amine oxidase; SAPD, 3-succinoylsemialdehyde pyridine dehydrogenase; HspA and HspB, 6-hydroxy-3-succinoyl pyridine hydroxylase.

The predominant nicotine-degrading Gram-negative *Pseudomonas *sp. followed pyrrolidine pathway [97]. However, the mechanism of this pathway has been studied poorly [98]. *Pseudomonas *sp. followed mainly four different pathways of pyrrolidine (Figure 2). Pyrrolidine pathway of nicotine degradation of *P. putida* S16 has been reported [28] (Figure 2). The intermediate metabolites and its nicotine-degrading genes of pyrrolidine pathways of other *Pseudomonas* sp. have not been fully elucidated or thoroughly characterized. Ruan et al. [26] reported that the *Pseudomonas* sp. HF-1 followed three other incomplete pyrrolidine pathways such as nicotine to cotinine, nicotyrine, and nornicotine (Figure 2). However, *Pseudomonas *sp. Nic22 degraded nicotine via cotinine and nornicotine pathways of pyrrolidine [29].

The pyrrolidine pathway of *P. putida *S16 initially attacked the pyrrolidine ring of nicotine to give *N*-methylmyosmine by the formation of a double bond. NicA enzyme was involved in the first step of dehydrogenation and it was believed that *nicA* gene encodes nicotine oxidoreductase that plays an important role in the initial steps of pyrrolidine pathway and involved in the degradation of nicotine to SP [99]. The reversible second step was carried out in the presence of water and a double bond of *N*-methylmyosmine was spontaneously hydrolyzed to form pseudooxynicotine, a direct precursor of a potent tobacco-specific lung carcinogen [99–101]. This carcinogenic intermediate metabolite pseudooxynicotine was further dehydrogenated to give methylamine and SP by NicA enzyme. Tang et al. [99] hypothesized that two unstable compounds were produced during the conversion of pseudooxynicotine to SP. The 6th position of carbon of SP was further hydrolyzed to yield HSP by an unknown enzyme. The *hsp* gene encodes an enzyme 6-hydroxy-3-succinoyl pyridine hydroxylase (HSP hydroxylase). The *hsp* gene attacked on the 3rd position of HSP to form 2,5-DHP and succinic semialdehyde (Ssa) [28, 97, 102]. The 2,5-DHP was further degraded to maleamic acid, which deaminates by hydrolysis and produces maleic acid [103]. The cleavage of 5th and 6th position of carbon of maleic acid gave pyruvic acid, which entered into citric acid cycle [102]. On the other side, Ssa easily converted into succinic acid by succinic semialdehyde dehydrogenase (SsaDH) enzyme that is widely present in *Pseudomonas *sp. [104–106].

Interestingly, the two genes *hspA* and *hspB* that encode HSP hydroxylase are involved in the conversion of HSP to 2,5-DHP [97, 99, 107]. The hydroxylase enzyme is involved in oxidation reactions in which one of the two atoms of molecular oxygen is integrated into the substrate and another is used to oxidize NADH or NADPH [108–110]. In *P. putida *S16 4879 bp *nic* gene cluster encoded three open reading frames (ORF), namely, ORF1 (1853 bp), ORF2 (936 bp), and ORF3 (582 bp). The ORF1 encoded *nicA* gene and ORF2 encoded *hspA* gene, whereas the function of remaining ORF3 is unknown [97, 99, 107]. Nevertheless, the newly identified *hspB* gene was located on 30 Kb of DNA away from the *nic* gene cluster [107]. The molecular mass of HspA has 38 kDa and NADH dependent, while HspB has 40 kDa, a dimer, and a prosthetic group FAD dependent. The deletion of *hspB* gene in the mutant strain could not degrade HSP, suggesting that *hspB* gene plays an important role in the conversion of HSP to 2,5-DHP [107]. The molecular mass of flavin adenine mononucleotide (FMN) dependent enzyme NicA is approximately 65 kDa that degraded nicotine into *N*-methylmyosmine, pseudooxynicotine, and SP in *P. putida *S16. These three nicotine intermediate metabolites were confirmed by electrospray ionization quadrupole time of flight mass spectrometry (ESI-Q-TOF-MS) analysis [99]. All three genes have been cloned and overexpressed in *Escherichia coli *[97, 99, 107]. However, the *Pseudomonas *sp. Nic22 bacteria followed other pyrrolidine pathways that produced myosmine, 2,3′-dipyridyl, and cotinine during biodegradation of nicotine [29] (Figure 2). *Shinella *sp. HZN1 produced three nicotine intermediate metabolites during biodegradation, which were characterized and identified as cotinine, myosmine, and nicotyrine using gas chromatography-mass spectrometry (GC-MS) analysis [36]. Four major nicotine intermediate metabolites pseudooxynicotine, SP, an unstable compound 3-succinoylsemialdehyde pyridine (SAP), and HSP and three nicotine-degrading genes *sir A2*, *pao*, and *sap *were identified in *Pseudomonas *sp. HZN6 [35, 98]. Sir A2 protein was encoded by *Sir A2* and a sulfurtransferase homologue gene is responsible for the degradation of SP [98]. An unstable nicotine intermediate metabolite SAP produced during the conversion of pseudooxynicotine by pseudooxynicotine amine oxidase (PNAO) was encoded by *pna *gene. This enzyme oxidatively deaminates pseudooxynicotine by noncovalently bound FAD, O_2_, and H_2_O and forms SAP, methylamine, and H_2_O_2_. Another gene *sap *encodes NADP^+^ dependent enzyme 3-succinoylsemialdehyde pyridine dehydrogenase (SAPD) dehydrogenates SAP to SP [35]. Another nicotine-degrading bacterium *Pseudomonas *CS3 produced three new nicotine intermediate metabolites [111] (Figure 2). In the initial step, demethylation of nicotine forms a metabolite 3-(3,4-dihydro-2H-pyrrol-5-yl) pyridine. Concurrently, further degradation was initiated by hydroxylation of 2nd position of pyrrolidine ring of nicotine to form 1-methyl-5-(3-pyridyl) pyrroline-2-ol which is further transformed to cotinine.

Wang et al. [112] identified nicotine-degrading gene *hsp* in the plasmid pMF1 (21 Kb) of *Pseudomonas *sp. HF-1 and reported that nicotine degradation is regulated by plasmid not chromosomal DNA. Ketopantoate hydroxymethyltransferase encoded by *panB* gene of *P. putida *J5 is involved in nicotine catabolism. Pyruvic acid the end product of pyrrolidine pathway is a precursor of ketoisovalerate, which undergoes catabolism using the enzyme ketopantoate hydroxymethyltransferase leading to synthesis of vitamin pantothenate [113].

**Figure 3:**
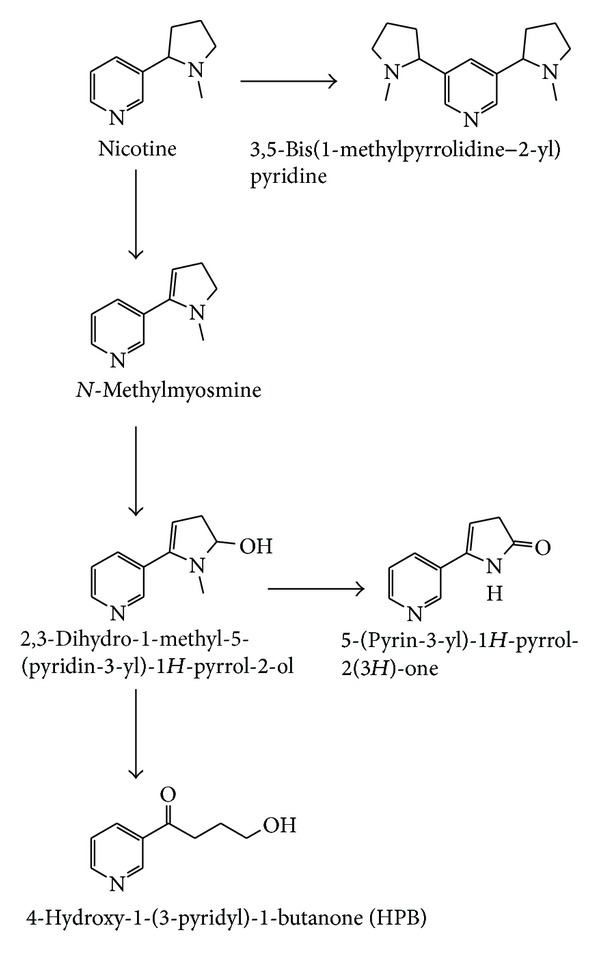
A variant pyrrolidine pathway of nicotine biodegradation by *P. plecoglossicida *TND35.

Raman et al. [2] recently reported that *P. plecoglossicida *TND35 followed a variant of pyrrolidine pathway, which is different from pathways of other bacteria and fungi (Figure 3). Strain TND35 oxidized pyrrolidine ring moiety of nicotine to form *N*-methylmyosmine. This intermediate was further hydroxylated at 2nd position of pyrrolidine ring to form a new cotinine analogue metabolite, 2,3-dihydro-1-methyl-5-(pyridin-3-yl)-1*H*-pyrrol-2-ol (IM2). In addition, this metabolite was demethylated and hydroxyl group of 2,3-dihydro-1-methyl-5-(pyridin-3-yl)-1*H*-pyrrol-2-ol was further oxidized to form another new cotinine analogue metabolite, 5-(pyridin-3-yl)-1*H*-pyrrol-2(3*H*)-one (IM3). Concurrently, 2,3-dihydro-1-methyl-5-(pyridin-3-yl)-1*H*-pyrrol-2-ol was further oxidized and released methylamine. This reaction further leads to opening of the pyrrolidine ring to form an end product 4-hydroxy-1-(3-pyridyl)-1-butanone (IM4). Interestingly, new metabolite 3,5-bis (1-methylpyrrolidin-2-yl) (IM5) was observed during nicotine degradation. This may be due to the cleavage of bond between pyridine and pyrrolidine ring of the nicotine. This *N*-methyl pyrrolidine ring attacked the pyridine moiety of nicotine and formed this metabolite.

### 4.3. Variant Pathway of Pyridine and Pyrrolidine for Nicotine Biodegradation

**Figure 4:**
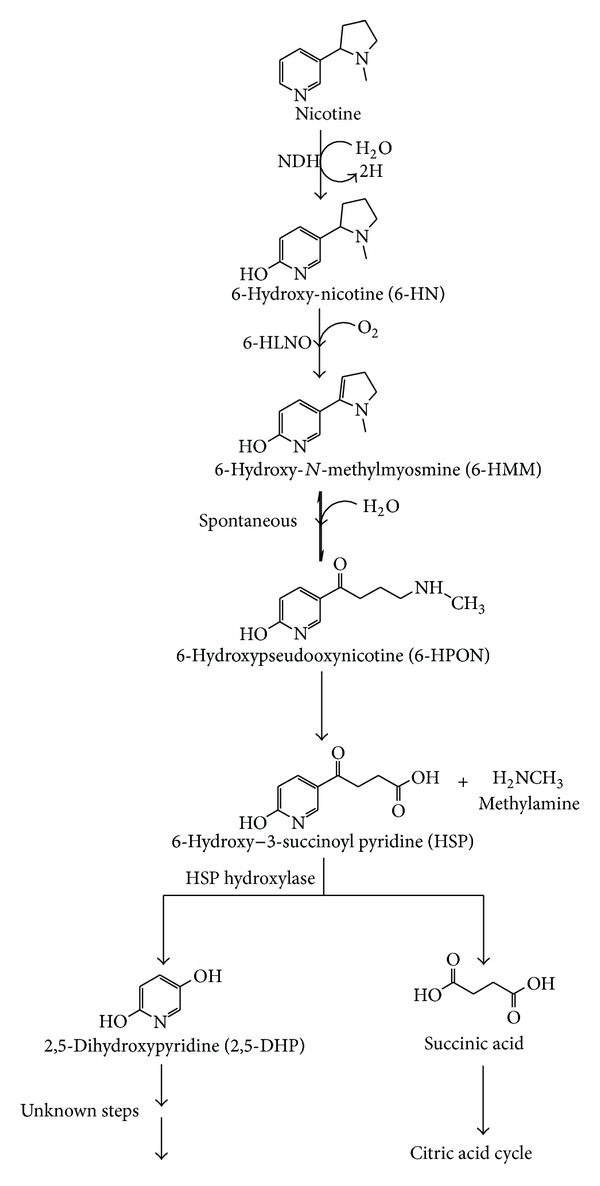
A variant of pyridine and pyrrolidine pathway of nicotine degradation by *A. tumefaciens *S33. NDH, nicotine dehydrogenase; 6-HLNO, 6-hydroxy-L-nicotine oxidase; HSP hydroxylase, 6-hydroxy-3-succinoyl pyridine hydroxylase.

The bacterium *A. tumefaciens *S33 followed a variant of pyridine and pyrrolidine pathway (Figure 4). This bacterium partially shared both the pathways and produced its intermediate metabolites [114]. Similarly, this bacterium also produced bright green colour initially and oxidized to brown colour at concentration above 3 g/L nicotine. The production of colour depends upon the concentration of nicotine, oxygen content, and pH of the medium [114]. Pathway of pyridine intermediate metabolites 6-HN, 6-HMM, and 6-HPON and pyrrolidine pathway metabolites HSP and 2,5-DHP were identified by ultraviolet-visible (UV-Vis) spectroscopy, thin layer chromatography (TLC), high performance liquid chromatography (HPLC), gas chromatography-high resolution-mass spectrometry (GC-HR-MS), and ESI-Q-TOF-MS analyses during nicotine degradation of *A. tumefaciens *S33. The catabolic nicotine-degrading enzymes NDH, 6-HLNO, and HSP hydroxylase were also observed in this bacterium. The initial step of pyridine pathway followed hydroxylation of nicotine to 6-HN by NDH. This metabolite was further oxidized and transformed into 6-HMM by 6-HLNO. The occurrence of spontaneous hydrolysis of 6-HMM into 6-HPON makes entry into pyrrolidine pathway. The metabolite 6-HPON is dehydrogenated and spontaneous hydrolysis leads to the production of HSP and removal of methylamine. However, similar mechanism was also observed during catabolic degradation of chlorine and aromatic amine, 2-phenethylamine [115, 116]. The HSP was further catabolized into 2,5-DHP. In contrast, *N-*methylmyosmine, pseudooxynicotine, and SP were not found during this degradation [114].

### 4.4. Demethylation Pathway for Nicotine Biodegradation

**Figure 5:**
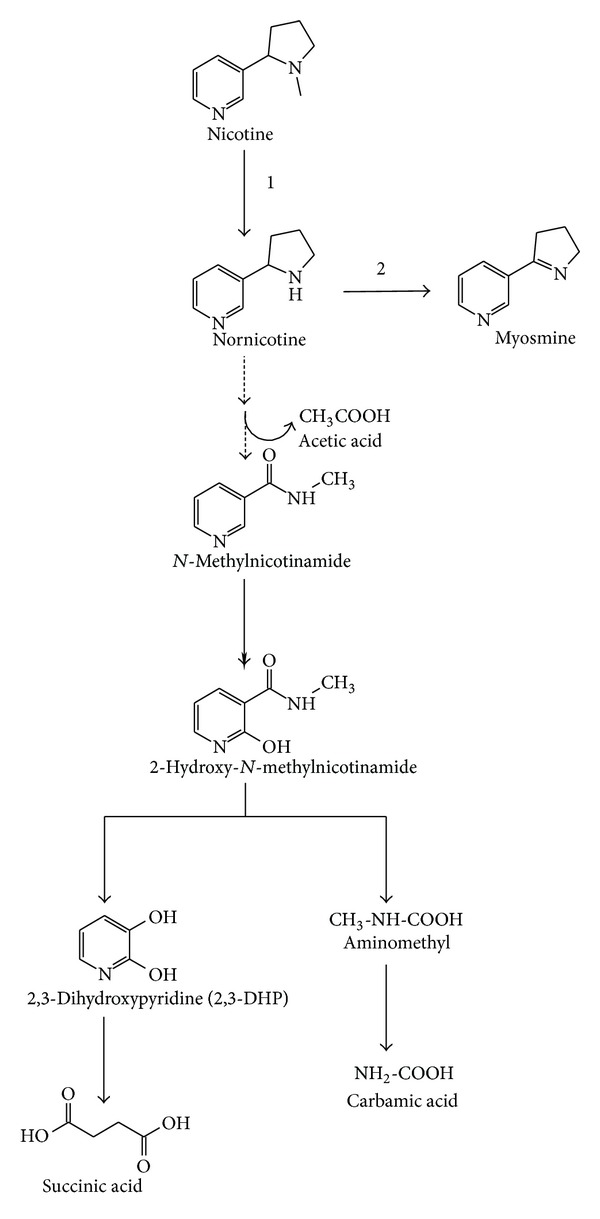
Demethylation pathway of nicotine degradation by fungi. 1. *A. oryzae *11282; 2. *M. gypseum*, *P. filamentosa *JTS-208, and *C. echinulata*.

Fungi such as *M. gypseum*, *P. filamentosa *JTS-208, and *C. echinulata *have been reported for nicotine degradation. These fungi are involved in the initial step, demethylation of nicotine that leads to the formation of nornicotine [21, 117] (Figure 5). Recently, Meng et al. [22] reported nicotine intermediate metabolites, namely, nornicotine, N-methylnicotinamide, 2,3-DHP, 2-hydroxy-*N*-methylnicotinamide, acetic acid, carbomic acid, and succinic acid on the basis of TLC, GC-MS, nuclear magnetic resonance (NMR), and Fourier transform infrared (FT-IR) analyses and proposed a hypothetical demethylation pathway of nicotine degradation by *A. oryzae *112822 (Figure 5). The primary step of this hypothetical pathway is the elimination of the removal of methyl group in the pyrrolidine ring of nicotine to form nornicotine, which is further converted into myosmine by the formation of double bond in the pyrrolidine ring (Figure 5). The subsequent cleavage of pyrrolidine ring resulted in the formation of unknown intermediate metabolite. The hydrolytic attack on the postulated unknown intermediate metabolite resulted in *N*-methylnicotinamide and acetic acid. The *N*-methylnicotinamide was hydroxylated to form 2-hydroxy-*N*-methylnicotinamide, which was catabolized to a new nicotine intermediate metabolite 2,3-DHP with the formation of aminomethyl. The aminomethyl was further transformed into carbomic acid. The opening of ring in 2,3-DHP leads to the formation of succinic acid, which enters into citric acid cycle [22]. Nevertheless, no catabolic enzymes were identified during the degradation of nicotine by fungi.

## 5. Biotechnological Applications of Nicotine Intermediate Metabolites

Biotransformation or biocatalysis involves the use of microorganisms to catalyze the conversion of one metabolite into another. These metabolites were catalyzed by whole microbial cells, cellular extracts, or enzymes [118]. Biotransformation is a promising tool, used in the synthesis of bulk chemicals for synthesis of pharmaceutical, food and agrochemical ingredients in the industry [119]. Nicotine is used as a starting material for the biocatalytic production of functionalized pyridines from renewable sources [120]. The easiest and friendliest ways of biotransformation approach were used to transfer toxic nicotine into valuable compounds such as HSP and DHP [110, 119, 121, 122]. Biotransformation intermediates of nicotine are widely used in anticancer therapies, antimalarial and analgesics drug development, and treatment of Parkinson's disease, hypertension and disorders of central nervous system [123]. Nicotine intermediate metabolites are precursors in the synthesis of drug such as analogues of epibatidine, an extremely effective analgesic molecule that is used in pharmaceutical industry [121]. Hydroxylated pyridine intermediates are used as precursors for the synthesis of drugs and instecticides via chemical methods [114]. The biologically active metabolites, 2,5- or 3,5-disubstituted pyridines, are catabolized from 6HLN and HSP, which is used for the synthesis of insecticide imidacloprid; SIB-1508Y is an anti-Parkinson's agent [119, 121, 122]. The important nicotine intermediate metabolite, 2,5-DHP, can be used as the initial material for the chemical synthesis of universal precursor, aminolevulinic acid. This precursor is used to synthesize plant growth regulators, herbicides, and drugs used in cancer therapies and to synthesize porphyrins such as heme and chlorophyll [110]. The biotransformation nicotine intermediate metabolite HPB has been widely used as a biomarker for tobacco related lung cancer studies [2].

## 6. Conclusions and Future Perspectives 

Environmental pollution is one of the major problems in the world. Tobacco industries produced enormous amount of nicotine. The nonreadily degradable nicotine causes environmental problems and human health when directly entered into soil. Major microbes degrade the toxic compound, nicotine. In this paper, we have discussed all the metabolic pathways and the genes involved in nicotine degradation. These microbes produce various intermediate metabolic compounds of pharmaceutical importance during nicotine degradation. Bioremediation is one of the promising tools used to convert the toxic compounds into valuable compounds. These nicotine-degrading microbes can be used for bioremediation of nicotine-polluted environments. Large scale production of these intermediate metabolites of nicotine could be of great use in pharmaceutical industries.

## Figures and Tables

**Table 1:** The optimal conditions, nicotine degradation efficiency, and degradation pathways of nicotine-degrading bacteria.

Microorganism	Medium	Optimal conditions (pH and Temperature)	Nicotine degradation efficiency (g/L)	Pathway	Reference
Fungi					
*Pellicularia filamentosa* JTS-208	Synthetic	ND	0.04/20 d	Demethylation	[21]
*Cunninghamella echinulata *IFO-4444	Synthetic	ND	0.54/13 d	Demethylation	[21]
*Aspergillus oryzae *112822	Synthetic	6.5, 28°C	2.12/40 h	A variant of pyridine and pyrrolidine	[22]
Gram-positive bacteria					
*Arthrobacter nicotinophagum *N. sp.	Synthetic	6.8 to 7.0, 20 to 25°C	1.62/56 h	Pyridine	[23]
*Arthrobacter *sp. HF-2	Synthetic	7.0, 30°C	0.70/43 h	ND	[24]
Gram-negative bacteria					
*Pseudomonas* sp. 41	Synthetic	6.4, 30°C	1.30/24 h	Pyrrolidine	[25]
*Pseudomonas* sp. HF-1	Synthetic	6.5 to 7.5, 30°C	1.30/25 h	Pyrrolidine	[26]
*Ochrobactrum intermedium* DN2	Synthetic	30°C	0.50/36 h	ND	[27]
*P. putida* S16	Synthetic	7.0, 30°C	3.00/10 h	Pyrrolidine	[28]
*Pseudomonas* sp. Nic 22	Synthetic	6.5, 30 to 34°C	2.89/52 h	ND	[29]
*P. putida* J5	Synthetic	30°C	3.97/48 h	ND	[30]
*Rhodococcus* sp. Y22	Synthetic	7.0, 28°C	1.00/52 h	ND	[31]
*Agrobacterium tumefaciens *S33	Synthetic	7.0, 30°C	5.00/18 h	A variant of pyridine and pyrrolidine	[32]
Gram-negative bacteria					
*Ensifer *sp. N7	Synthetic	7.0, 28°C	3.35/48 h	ND	[33]
*Pseudomonas* sp. ZUTSKD	Synthetic	7.5, 30°C	1.55/12 h	ND	[34]
*Pseudomonas sp. *HZN6	Synthetic	7.0, 30°C	0.50/12 h	Pyrrolidine	[35]
*Shinella* sp. HZN1	Synthetic	7.0, 30°C	0.50/9 h	ND	[36]
*Acinetobacter* sp. TW	Synthetic	7.0, 30°C	1.00/12 h	ND	[37]
*Sphingomonas* sp. TY	Synthetic	7.0, 30°C	1.00/18 h	ND	[37]
*Pseudoxanthomonas* sp. 5–52	Synthetic	7.0, 28°C	1.00/28 h	ND	[38]
*P. stutzeri *	Synthetic	7.5, 37°C	2.50/36 h	ND	[39]
*Sinorhizobium* sp. 5–28	Synthetic	7.0, 28°C	1.50/28 h	ND	[38]
*Ochrobactrum* sp. 4–40	Synthetic	7.0, 28°C	0.50/28 h	ND	[38]
*P. plecoglossicida *TND35	Synthetic	7.0, 30°C	3.0/12 h	A variant of pyrrolidine	[2]

ND: not detected.

**Table 2:** The nicotine degradation efficiency of different nicotine-degrading bacteria on tobacco leaves and tobacco wastes.

Bacterium	Source	Duration (time/temperature)	Nicotine (mg/g)	Nicotine degradation (%)	Reference
*Micrococcus nicotianae *	Tobacco leaves	NM	NM	0.83	[40]
*Debaryomyces nicotianae *	Tobacco leaves	NM	NM	0.45	[40]
*Cellulomonas* sp.	Tobacco leaves	NM	NM	15.00	[41]
*A. oxidans α*-2	Tobacco waste	212 h	700	NM	[7]
*A. oxidans* pAO1	Tobacco waste	125 h	3400	NM	[7]
*P. putida *	Tobacco waste	40 h	3300	NM	[7]
*P. putida* J5	Tobacco leaves	7 d	2.8	11.72	[30]
*Pseudomonas* sp. Nic 22	Tobacco leaves	30°C	NM	33.10	[29]
*Ensifer* sp. N7	Tobacco leaves	NM	4.1	16.00	[42]
*Arthrobacter* sp.	Tobacco leaves	NM	NM	28.00 to 39.00	[33]
*Pseudoxanthomonas* sp. 5–52	Tobacco leaves	NM	NM	47.20	[38]
*Sinorhizobium* sp. 5–28	Tobacco leaves	NM	NM	72.50	[38]
*Ochrobactrum* sp. 4–40	Tobacco leaves	NM	NM	51.50	[38]
*P. stutzeri* ZCJ	Tobacco leaves	7 d/37°C	NM	32.24	[39]
*P. plecoglossicida *	Tobacco leaves	10 h	13	88.00	[2]
*P. plecoglossicida *	Tobacco wastes	10 h	5	96.10	[2]

NM: not mentioned.
